# Q&A: what are brassinosteroids and how do they act in plants?

**DOI:** 10.1186/s12915-016-0340-8

**Published:** 2016-12-22

**Authors:** Jiao Tang, Zhifu Han, Jijie Chai

**Affiliations:** 1 Key Laboratory of Protein Sciences (Tsinghua University), Ministry of Education, Beijing, 100084 China; 2 Tsinghua-Peking Center for Life Sciences, Beijing, 100084 China; 3 Institute of Apicultural Research, Chinese Academy of Agricultural Sciences, Beijing, 100093 China

## Abstract

Brassinosteroids (BRs) are a class of polyhydroxylated steroidal phytohormones in plants with similar structures to animals’ steroid hormones. Brassinosteroids regulate a wide range of physiological processes including plant growth, development and immunity. Brassinosteroid signalling and its integration with other signalling pathways have been investigated thoroughly at the molecular level.

## What are the plant steroid hormones brassinosteroids?

Brasinosteroids, defined as the sixth plant hormone after the classic plant hormones auxin, gibberellins, cytokinin, abscisic acid and ethylene, are analogous to animal steroid hormones in structure [1, 2]. Similar to animal hormones, brassinosteroids play crucial roles in diverse aspects of plant biology, including cell elongation, cell division, root growth, photo-morphogenesis, stomatal and vascular differentiation, seed germination, immunity and reproduction [3, 4]. Brassinosteroids are also involved in regulating the metabolism of plant oxidation radicals, ethylene synthesis and root gravitropic response, and have a role in mediating plant responses to stress, such as freezing, drought, salinity, disease, heat and nutrient deficiency [5–7]. This subfamily of hormones regulates a broad range of processes in plant development and responses to environmental stresses, and their analogs have been shown to bring substantial increases in grain yield, depending on growth status.

## How were brassinosteroids found?

Mitchell and co-workers extracted specific ingredients with growth-promoting activity from *Brassica* pollen after screening nearly 60 species of plants. Histological analysis showed that the reactions induced by these ingredients were different from those induced by gibberellins [8]. They therefore speculated that these ingredients were a new class of hormones, termed brassins. This hypothesis, however, was not accepted by some other researchers, who argued that the physiological activities of the ingredients could have been caused by gibberellin due to the crude nature of the extract from which brassins were identified [9]. Given the potential applications of brassins in agriculture, efforts organized by the US Department of Agriculture led to purification of 4 mg of brassins from 500 pounds of bee-collected *Brassica* pollen. The crystal structure of the purified brassins was then solved and brassinolide was identified as the active component [10]. These findings marked the discovery of the first plant steroidal hormone. Currently, nearly 70 kinds of natural brassinolide analogues have been isolated from tissues of various plant species, which compose the new class of plant hormone, BRs [11].

## What are the structures of brassinosteroids?

**Fig. 1:**
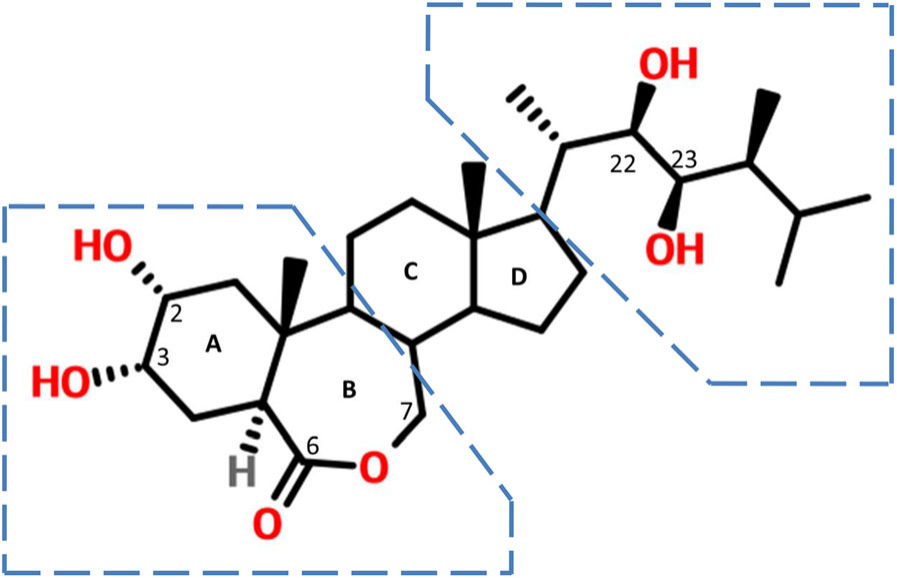
The chemical structure of brassinolide (BL) with the steroid rings labelled as *A*, *B*, *C* and *D*. The parts within the dashed lines can be substituted by different groups, as described in the text

Brassinosteroids are classified as C_27_, C_28_ or C_29_ based on different alkyl-substitution patterns of the side chains. In general, a trans-fused A/B ring system with two hydroxyl groups at ring A and a 6-ketone or 7-oxa-6-ketone system at ring B are required for active BRs [1]. The chemical structure of brassinolide ((22R, 23R, 24S)-2a, 3a, 22, 23-tetrahydroxy-24-methyl-B-homo-7-oxa-5a-cholestan-6-one) [10], the most active BR identified thus far, is shown in Fig. 1. Other BRs differ from BL within the boxed area (a) and (b) on the basis of a 5α-cholestane skeleton [12] (Fig. 1). Additionally, BR conjugated forms with sugars or fatty acids have also been found, which are the inactive products of BR metabolism [12].

## What parts of plants are brassinosteroids usually found in?

Brassinosteroids have been found in various species of plants, including monoplast freshwater algae and brown algae, indicating that BRs are a widespread ancient plant hormone [11]. Distributions of BRs differ among distinct tissues of individual species. Pollens, immature seeds, roots and flowers were found to have the highest content, ranging from 1–100 ng/g fw (fresh weight), while shoots and leaves have lower amounts 0.01–0.1 ng/g fw [13]. The content and distributions of different BR analogues also vary among tissues. Unlike other hormones, endogenous BRs do not move between tissues but function in a paracrine or autocrine way as demonstrated by grafting experiments using a BR-deficient mutant of pea [14, 15]. One reason for this is likely that BR biosynthesis genes are widespread in various tissues of plant and BRs can be synthesized in situ. Long-distance effects of BRs depend on their crosstalk with other hormones like auxins and gibberellin [16]. However, BRs need transporting from their synthesis sites in the ER to the plasma membrane and early endosomal compartments where they are perceived through passive or active intracellular transport.

## What do plants do with excess brassinosteroids?

Plants synthesize BRs continuously to meet their need for growth and development, but excess BRs can be metabolized rapidly, as demonstrated by exogenous application of BRs. BR metabolism can be classified into modification of their steroidal skeletons and modification of their side chains [17]. A number of reactions, such as dehydrogenation, demethylation, epimerization, esterification, glycosylation, hydroxylation, side-chain cleavage and sulfonation, have been found to inactivate BRs, though the mechanisms underlying this remain unclear. It has been suggested that inactive BRs can be converted into active forms to maintain BR homeostasis [12, 18].

## What are the receptors for brassinosteroids?

Studies from several laboratories contributed to the finding of the first BR receptor [19]. Clouse et al. identified the first BR-insensitive (BRI) mutant (named *bri1*) by observing the promotion of root elongation under inhibitory concentrations of BR compared to the wild type in *Arabidopsis* [20]. The *bri1* mutant displayed dwarfism, reduced cell elongation, dark-green and thickened leaves, reduced apical dominance, delayed blooming and senescence, altered vascular patterning and male sterility. The positional cloning of BRI1 was performed by Jianming Li and J. Chory, who identified 18 alleles of *bri1*. Despite structural similarity between BRs and animal steroid hormones, BRI1 does not structurally resemble the nuclear steroid receptors of animals but encodes a leucine-rich repeat receptor-like kinase (LRR-RLK) with an extracellular leucine-rich repeat (LRR) domain and an intracellular serine/threonine kinase domain [21]. BRI1 is highly conserved across different plant species [19], consistent with the finding that BRs are widely present in plants. There are three BRI1 homologues in *Arabidopsis*, BRL1, BRL2 and BRL3. BRL1 and BRL3, but not BRL2, were shown to bind BRs with high affinity and rescue the phenotypes of the BRI1 mutation when expressed using the promoter of BRI1 [22]. Thus far, the ligands BRL2 might recognize still remain unknown. BRI1 is highly expressed in various tissues of plants and functions as the major receptor of BRs, whereas the expression of BRL1 and BRL3 is confined to vascular cells and display weak phenotypes when knocked out [22].

## What are the BRI1-associated receptor kinases?

In 2002, J.C. Walker’s group, using a *bri1* suppressor screen, and Jianming Li’s group, using a yeast two-hybrid screen, independently found a BRI1-interacting partner, an LRR-RLK named BRI1-associated kinase 1 (BAK1). Both groups presented evidence showing that BRI1 and BAK1 interacted with each other in vitro and in vivo, which contributed to BR signalling [23, 24]. BAK1 is also called SERK3, as it belongs to the family of SOMATIC EMBRYOGENESIS RECEPTOR-LIKE KINASEs (SERKs), of which there are five members (SERK1–SERK5) in *Arabidopsis* [25]. Later on, SERK1 and SERK4 were shown to have a similar role to BAK1/SERK3 in BR-induced signalling [26, 27]. The *serk1 bak1 bkk1* triple null mutant phenocopied a null *bri1* mutant, establishing an indispensable role of SERKs in BR signalling [27].

## How does BRI1 recognize brassinosteroids and consequently become activated?

**Fig. 2:**
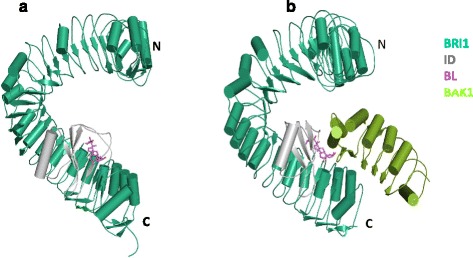
Brassinolide induces BRI1 heterodimerization with SERKs. **a** Overall structure of BL bound by BRI1LRR (*green*) shown as a structure graphic. BL (*pink*) is shown as a stick diagram. The island domain of BRI1 is shown in *grey*. ‘*N*’ and ‘*C*’ represent the N-terminal and C-terminal side of BRI1LRR, respectively. **b** Crystal structure of BRI1LRR-BL-BAK1LRR complex. BAK1LRR is shown in *pale green*

In 2011, two groups provided structural and biochemical data demonstrating that the extracellular LRR domain of BRI1 (BRI1LRR) was sufficient for recognition of BL [28, 29]. Structural analyses revealed that a hydrophobic groove, formed between the inner surface of the helical BRI1LRR and an embedded island domain, is responsible for the specific recognition of BL by BRI1 (Fig. 2). This mechanism is conserved in the recognition of BL by BRL1 [30] and likely BRL3 given the highly conserved amino acids of the BL-binding groove among the three BR receptors. Two years later, the same two groups presented the crystal structures of BRI1LRR-BL-BAK1LRR/SERK1LRR ternary complexes [31, 32]. Coupled with biochemical data, these structures demonstrated that BL induced a heterodimeric BRI1LRR-BAK1/SERK1LRR complex for BRI1 activation (Fig. 2). These results indicate that ligand-induced dimerization seen for the activation of receptor tyrosine kinases (RTKs) and other single trans-membrane receptors in animals is also conserved in the activation of plant RLKs [33–36]. Furthermore, BAK1 or SERK1 in the complex is directly involved in recognition of BL, forming one heterodimerization interface between BRI1LRR and BAK1LRR/SERK1LRR [31, 32]. These results indicate that BAK1/SERK1 function as co-receptors with BRI1 for BR-induced BRI1 activation.

## How are the signals downstream of brassinosteroid signalling transduced?

**Fig. 3:**
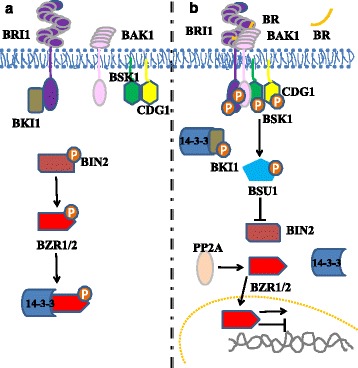
The brassinosteroid signal transduction pathway in the absence (**a**) and presence (**b**) of brassinosteroid (BR) in *Arabidopsis*. Brassinosteroid binding induces BRI1 heterodimerization with its co-receptor BAK1, resulting in activation of BRI1, phosphorylation of BKI1 by BRI1 and dissociation of BKI1 from BRI1. These events lead to full activation of BRI1, which phosphorylates BSK1 and CDG1. Once activated, BSK1 and CDG1 promote the activation of the phosphatase BSU1, thus dephosphorylating and inactivating BIN2. The inactivated BIN2 allows the presence of the dephosphorylated form of BZR1/2, which can move into the nucleus and regulate transcription of many target genes. The phosphorylated BZR1/2 are retained in the cytoplasm by 14-3-3. *Arrows* and *short lines* represent promotion and inhibition, respectively. The small circles containing the letter ‘*P*’ indicate phosphorylation

The BR signalling pathway is a complex phosphor-relay system that mediates plant growth and development [37] (Fig. 3). BR binding induces BRI1 phosphorylation of BRI1-KINASE INHIBITOR 1 (BKI1), thus relieving BKI1 inhibition of BRI1 and fully activating BRI1 through mutual phosphorylation between BRI1 and BAK1 or other SERK members. Once activated, BRI1 phosphorylates the BR-SIGNALING KINASE (BSK1) and CONSTITUTIVE DIFFERENTIAL GROWTH 1 (CDG1) kinase, which in turn activate a PP1-type phosphatase named BRI1-SUPPRESSOR 1 (BSU1). The activated BSU1 and PROTEIN PHOSPHATASE 2A (PP2A) dephosphorylate and inactivate the GSK3-like kinase named BRASSINOSTEROID INSENSITIVE 2 (BIN2), allowing BR response transcription factors BRASSINAZOLE RESISTANT 1/2 (BZR1/2) to accumulate in the nucleus and bind to DNA. Additionally, many other transcription factors also contribute to the global BR responses [38–40].

## The potential role of brassinosteroids in the plant growth-immunity trade-off is intriguing—can you describe it?

Brassinosteroids regulate many development processes in plants, as well as responses to environmental stresses and their roles in the growth–defence trade-off have profound implications in agriculture and natural ecosystems. To ensure perpetuation, plants need to balance their limited resources for growth and defence [41]. Several plant hormones, including BRs, have been suggested to play roles in the trade-off between growth and defence [42]. Recent studies indicated that interaction of BR signalling with PAMP-triggered immunity is unidirectional and negative [43, 44]. More recent data appear to support the idea that the interaction is located at the transcriptional level rather than at the receptor complex [45–48], though the underlying mechanisms remain debatable. Understanding of the mechanisms for these trade-offs is expected to provide a foundation for development of breeding strategies to maximize crop yield.

## What other unknowns are there in brassinosteroid signalling?

Great advances have been achieved in our understanding of the BR signalling pathway and BR biosynthesis and metabolism. However, it remains unclear how brassinosteroid-induced BRI1-BAK1 heterodimerization activates the kinase activity of BRI1. Fully addressing this question will require detailed elucidation of structure of the full-length complex. New components are being identified in brassinosteroid signalling and understanding of how they are integrated with other signalling pathways will be important to gain a comprehensive and systematic view of brassinosteroid functions in various plants [49, 50]. A full elucidation of brassinosteroid signalling events and their integration with other signalling pathways will enable brassinosteroid to be applied in agriculture.
